# Innovations in TB Screening and Preventive Therapy Services for PLHIV in Yogyakarta City, Indonesia

**DOI:** 10.3390/tropicalmed10010028

**Published:** 2025-01-20

**Authors:** Dani Catrianiningsih, Guardian Yoki Sanjaya, Geoff Chan, Betty Weri Yolanda Nababan, Rina Triasih, Desthi Diah Intani, Endang Sri Rahayu

**Affiliations:** 1 Center for Tropical Medicine, Universitas Gadjah Mada, Yogyakarta 55281, Indonesia; bettynababan2014@gmail.com (B.W.Y.N.); rina_triasih@yahoo.com (R.T.); desthiintani2013@gmail.com (D.D.I.); 2 Department of Health Policy and Management, Faculty of Medicine, Public Health and Nursing, Universitas Gadjah Mada, Yogyakarta 55281, Indonesia; gysanjaya@ugm.ac.id; 3 TB Elimination and Implementation Science Group, Burnet Institute, Melbourne, VIC 2004, Australia; geoff.chan@burnet.edu.au; 4 Department of Child Health, Faculty of Medicine, Public Health and Nursing, Dr. Sardjito Hospital, University of Gadjah Mada, Yogyakarta 55281, Indonesia; 5 Yogyakarta City Health Office, Disease Control, Yogyakarta 55165, Indonesia; endangrahayu@ymail.com

**Keywords:** TB screened, TB prevention therapy, people living with HIV

## Abstract

Tuberculosis preventive therapy (TPT) for people living with HIV (PLHIV) has been recommended by Indonesia’s National TB Program since 2014 but has seen limited implementation. This study describes TB screening and TPT initiation from 2019 to 2022 among eight healthcare facilities supported by the Zero TB Yogyakarta (ZTB) project. ZTB assigned a dedicated nurse to assist with active TB screening among PLHIV and recommended the immediate initiation of TPT as an innovation implemented. Data were obtained from the national HIV program reporting system, routinely reported by ART clinics from 2019 to 2022. We conducted a descriptive analysis, comparing the pre-intervention and intervention periods. During the intervention, there was a significant increase in PLHIV visits to healthcare facilities where TPT eligibility was assessed. At health centers, TB screening coverage for PLHIV decreased toward the end of the baseline period but recovered during the intervention. The number of PLHIV starting TPT also rose during the intervention. While the direct impact of ZTB is difficult to measure, the changes observed indicate progress in integrating TB/HIV services and enhancing TB prevention among PLHIV. Ongoing support, training, and supervision of healthcare facilities are crucial for improving TB screening and TPT provision.

## 1. Introduction

According to the World Health Organization (WHO), tuberculosis (TB) is the leading cause of death among people living with HIV, accounting for approximately 167,000 deaths globally in 2022 [1]. Despite global efforts to tackle TB and HIV co-infection, TB–HIV co-infection remains a major health problem worldwide, particularly in low and middle-income countries [2]. The WHO recommends collaboration between TB and HIV services in primary healthcare facilities and hospital settings to reduce mortality due to both undiagnosed and untreated HIV among TB patients, and undiagnosed and untreated TB among people living with HIV (PLHIV). The integration of services aims to identify more individuals with TB–HIV coinfection so that prompt treatment can be administered [3]. The WHO recommended interventions for the collaboration between services in health facilities to manage TB among people known to have HIV including intensive case finding through TB screening in people living with HIV (PLHIV); administration of TB preventive therapy; and control of TB infection [3].

Routinely screening for TB in HIV patients can promptly detect TB infection and active TB disease, allowing for early treatment and reducing the risk of disease [4]. The TB screening algorithm for HIV patients may vary depending on resources and healthcare service contexts. However, many countries have adopted into their guidelines the 2011 WHO recommendation to screen for four symptoms at every visit using the WHO-recommended four-symptom screen (W4SS) [3,4,5,6]. The W4SS screens for cough (of any duration), fever, weight loss, and night sweats. PLHIV who screen positive should be tested with a WHO-recommended molecular rapid diagnostic test such as the GeneXpert MTB/RIF assay, while those who screen negative should be started on preventive therapy for TB. However, in PLHIV the sensitivity of W4SS followed by GeneXpert MTB/RIF is 0.58 with a specificity of 0.99 and could result in approximately one-third of screened PLHIV starting TPT when a full course of anti-TB treatment would be more appropriate [5,6,7].

Chest X-ray (CXR) can be used in conjunction with W4SS. Algorithms that use abnormal CXR and positive W4SS, were reported to have a sensitivity of 0.93 and a specificity of 0.20 among PLHIV [8]. Consequently, the WHO consolidated guidelines on tuberculosis—Module 2: Screening includes guidance that “Among adults and adolescents living with HIV, chest X-ray may be used to screen for TB disease” and the inclusion of CXR in TB screening among PLHIV would be expected to reduce the rate of false negatives but with the tradeoff of a higher rate of false positives for presumptive TB [3,8]. 

In addition to detecting active TB among PLHIV, administration of TPT is recommended as part of TB–HIV service integration [3,9]. The goal of TPT is to inhibit the progression from possible TB infection to active TB disease, thereby reducing the morbidity and mortality associated with TB—including the need for hospitalization—and ultimately decreasing TB transmission. The administration of TPT has been reported to reduce TB morbidity by 38% in conjunction with ART [7,10]. Moreover, in studies examining the effectiveness of TB prevention regimens in PLHIV, TPT is estimated to protect against TB incidence for a period ranging from 3 to 7 years. As such, TPT is recommended as an integral component of the standard service package for PLHIV, and an essential component of Antiretroviral Therapy (ART) services for managing HIV infection [11].

A crucial question is how to operationalize the recommended interventions for TB screening and TPT among PLHIV. Implementation models are needed to address context-specific challenges relating to implementation and coverage [12,13]. These challenges encompass the availability and competence of healthcare personnel; drug supply chains; diagnostic facilities; patient adherence to treatment regimens; the stigma endured by individuals affected by TB/HIV; and accurate documentation and reporting of program activities [14,15]. Furthermore, the onset of the COVID-19 pandemic introduced additional hurdles and difficulties in providing TB/HIV care services, highlighting the need to adapt models to emergent challenges [16,17].

In Indonesia, which is one of the 30 high-burden countries for TB and HIV-associated higher coverage of tuberculosis (TB), interventions for people living with HIV (PLHIV) is needed. Indonesia’s TB incidence is estimated at 354 per 100,000 individuals and it recorded a total of 427,201 HIV cases, with approximately 10,174 (2.4%) cases of co-infection with TB [18,19]. The National Tuberculosis Program (NTP) recommends that people living with HIV (PLHIV) undergo TB screening at every routine monthly visit for antiretroviral (ART) medication pick-up. However, as of 2020, only 80% of PLHIV had undergone TB screening, and only 12% of PLHIV had initiated tuberculosis preventive treatment (TPT). These figures fall short of the national targets of achieving 100% TB screening and 45% TPT initiation rates. The situation in Yogyakarta City mirrors the national pattern, where universal TB screening is not being conducted for the 1722 registered PLHIV, and the percentage of PLHIV visiting healthcare facilities remains below 100%. Moreover, the coverage of TPT is less than 40% in this context [20,21].

The Zero TB Yogyakarta project (ZTB) is a project that aims to support the health system in Yogyakarta to eliminate TB. One of the areas ZTB supports is the detection of TB among PLHIV and the initiation of TPT to prevent further TB disease. We describe the model that ZTB used to support increased coverage of TB screening and TPT among PLHIV in Yogyakarta City. As part of this model, we assigned a dedicated nurse to provide support to eight ART service facilities. The support included ensuring that PLHIV received regular TB screening and immediate TPT initiation for those eligible. The assigned nurse also assisted and ensured that data were reported to the national recording system. The aim of this study was to evaluate whether there were improvements in the provision and coverage of TB–HIV services during the intervention period compared to the baseline period, using routine surveillance data reported in Indonesia’s national HIV and AIDS information system (SIHA).

## 2. Materials and Methods

### 2.1. Study Design and Setting

This study was a part of ZTB, which is a collaborative program between the Center for Tropical Medicine at Universitas Gadjah Mada Yogyakarta Indonesia, and the Burnet Institute in Australia. In partnership with the Yogyakarta City Health Office Indonesia, ZTB has been implementing a comprehensive strategy for TB services since 2020. This strategy focuses on active case finding, effective treatment, and TB prevention. One component of the project has been developing and supporting the implementation of models to improve the coverage of TB screening and TB preventive therapy among PLHIV at ART facilities in Yogyakarta, Indonesia. 

Yogyakarta City is an urban area located in the Special Region of Yogyakarta, Indonesia. In 2021, the city had a population of 373,589 people, with an estimated 1722 cases of HIV and 96 cases (5.5%) of co-infection with TB. ZTB developed an innovative model of TB–HIV service support, which included the use of symptom-based and chest X-ray (CXR) examinations for TB screening, as well as the use of a dedicated nurse assigned to offer on-site support and technical assistance for organizing and reporting TB/HIV services. The TB–HIV innovation was initiated in 2021 at eight ART clinics: four were at primary health centers and four were at hospitals. This support was provided in coordination with the Yogyakarta City Health Office, doctors, and HIV program staff in the ART clinics. 

In this study, we evaluated the existing TB–HIV program by utilizing data processed from the national HIV reporting system (SIHA). The indicators used included the number of PLHIV who visited healthcare facilities and underwent TB screening, as well as those who initiated ART. The data were analyzed across two periods—before and after the intervention—to assess the changes resulting from the support provided by the ZTB project and to evaluate the effectiveness of the program. Table 1 shows the service model before the intervention (the routine TB–HIV program approach) and the ZTB-supported model.

#### 2.1.1. The Routine TB–HIV Program Approach

In healthcare facilities in Indonesia, standard TB screening for PLHIV is conducted according to the national TB program guidelines in Indonesia using W4SS (cough of any duration, fever, weight loss, and night sweats) [22]. Screening should be conducted at every drug collection visit or when PLHIV visits a health facility for any symptoms. PLHIV are considered to have presumptive TB if they report one or more symptoms. Those with presumptive TB are tested with a rapid molecular diagnostic test (GeneXpert MTB/RIF). The final decision is made by a doctor to determine the necessary subsequent interventions for the patient. If diagnosed with TB, the healthcare facility doctor initiates TB treatment. PLHIV are eligible for TPT if they meet the following criteria: (1) do not have active TB, (2) show no symptoms of TB, (3) have no contraindications to therapy, and (4) have not received TPT in the last three years. As per Indonesian guidelines, doctors prescribe TPT based on the patient’s antiretroviral regimen (ART) to ensure optimal treatment outcomes [23,24,25]. In 2020, there were two recommended regimens: six months of isoniazid, taken once a day for six months (6H); and a combination of rifapentine and isoniazid, taken once a week for three months (3HP). 3HP did not become available in healthcare facilities in Yogyakarta until 2022. Therefore, before 2022 eligible PLHIV were given 6H for TPT [3,26].

TB/HIV services were provided by a designated team of staff employed by each intervention healthcare facility. This team included a doctor overseeing medical care, healthcare staff involved in the HIV program, and a reporting and recording (RR) officer handling documentation and reporting duties. The responsibilities of HIV program staff included supporting patients to attend their follow-up appointments, monitoring their adherence to therapy, and implementing doctors’ recommendations. RR officers were responsible for maintaining patient registers and drug registers, which form the basis of reports to the national HIV recording system (SIHA) every month.

#### 2.1.2. The ZTB-Supported Model

##### Screening and Diagnosis for TB

ZTB conducted routine community-based active case finding (ACF) using mobile CXR in collaboration with primary health centers in Yogyakarta city. In this ACF, populations with a high risk of TB, including PLHIV, were invited to come to the mobile service and undergo TB screening using symptom screening and chest X-ray. In addition to community-based ACF, ZTB conducted dedicated screening days using mobile CXR for PLHIV. ART clinics assisted with inviting PLHIV to attend these screening days, which were held at the ART clinic or at community locales that were convenient for and acceptable to PLHIV. At these dedicated screening days, regardless of their symptoms, all PLHIV would undergo chest X-rays. PLHIV with a symptom or CXR suggestive of TB were requested to provide a spot sputum sample. At the end of each screening day, the collected spot sputum samples were transported to a district or provincial laboratory to be tested for GeneXpert MTB/RIF testing. PLHIV with a negative screening result was reviewed for eligibility to start TPT. If eligible, the TPT was initiated by the HIV clinics. PLHIV were eligible for TPT if they had not received TPT in the preceding three years. The final diagnosis was made by doctors at the HIV clinics supported by a pulmonologist and a pediatrician in a case discussion meeting.

##### TPT Services

PLHIV assessed as eligible for TPT (based on the Indonesian guidelines) either through routine screening at HIV clinic visits or through ACF for PLHIV were initiated on TPT at the health facility where they were registered as HIV patients. ZTB provided support for the procurement of 3HP and helped calculate the need for 3HP for the program—the proposed procurement of regiments to the nation through the health office. Treatment for TPT was monitored during follow-up visits at the treating health facility. Any side effects reported by or observed in PLHIV on TPT were managed by a doctor at the treating health facility. TPT outcomes were assigned based on whether the person completed the treatment, refused it, or was lost to follow-up.

A nurse was employed by ZTB to support and supervise the TB–HIV services at the study clinics. A number of HIV–TB activities were the responsibility of this nurse:

- Provide capacity building (training) to the study site nurses on TB–HIV services.
- Coordinate with the ACF team from Zero TB Yogyakarta when referring PLHIV for chest X-ray screening at mobile service.
- Coordinate with the Health Department and HIV program managers at each facility to determine schedules, locations, and PLHIV to be invited to a special ACF event.
- Support HIV clinics to report the data from these activities in the national HIV recording system (SIHA).
- Follow up on the results of the ACF activities: ensuring the data from ACF was given to the primary health centers.
- Ensure that eligible PLHIV received the treatment needed (anti-TB drugs or TPT) and the action to be reported in SIHA.

### 2.2. Data Collection

The individual monthly reports on TB–HIV activities for each intervention health facility were extracted from the SIHA. These reports are government-mandated for health facilities providing HIV services and are routinely entered by the facilities into SIHA. We collected the surveillance indicators relating to PLHIV who attended monthly HIV visits, PLHIV screened for TB, and TPT eligibility and initiation. We collected SIHA reports from January 2019 to December 2022 for the eight ART clinics that were supported by the ZTB project.

### 2.3. Data Analysis

The raw exports from SIHA, received as individual reports for each health facility and each month, were merged into a single data set using R version 4.2.2 (R Foundation for Statistical Computing, Vienna, Austria); Stata version 17 (StataCorp, College Station, TX, USA) for intermediate data cleaning and processing; and R for final analysis of the processed data. The SIHA report data were divided into two periods based on date: a pre-intervention period (from January 2019 to December 2020); and an intervention period (January 2021 to December 2022). To facilitate data analysis, for each of the included indicators, we aggregated the monthly data into quarterly totals. We also categorized it into a health facility type (primary health center or hospital) based on the government classification of the health facility that reported the data. We assessed data completeness for each monthly report based on whether a value was reported for each indicator. Indicator data could be missing if a monthly report was submitted without data for that indicator, or if the monthly report was not submitted. The proportion of missing indicator data was calculated as the number of submitted monthly reports with indicator data reported divided by the total number of months for the study period.

We conducted a descriptive analysis of TB screening and TB preventive therapy (TPT) indicator data for the pre-intervention and the intervention periods with disaggregation by health facility type. Screening coverage was calculated as the proportion of patient visits for ART at which TB screening was conducted. Screening coverage was summarized by calculating the median and interquartile range (IQR) for each calendar quarter throughout the study for the two periods. TB preventive therapy could not be calculated because of the way that these indicators are reported. In SIHA, the number of patient visits at which a patient was assessed as eligible for TPT can include multiple ART visits from the same individual before they start TPT. Hence, for TPT indicators, we restricted our analysis to the reported counts of ART visits at which TPT eligibility was assessed and counts of TPT initiations. 

## 3. Results

### 3.1. Data Availability

Between January 2019 and December 2022, there were 378 reports for the eight ART facilities available in SIHA, compared to an expected 384 reports (8 facilities × 48 months). For the SIHA indicators used in this study, Table 2 shows the number of reports where data were available.

**Figure 1:**
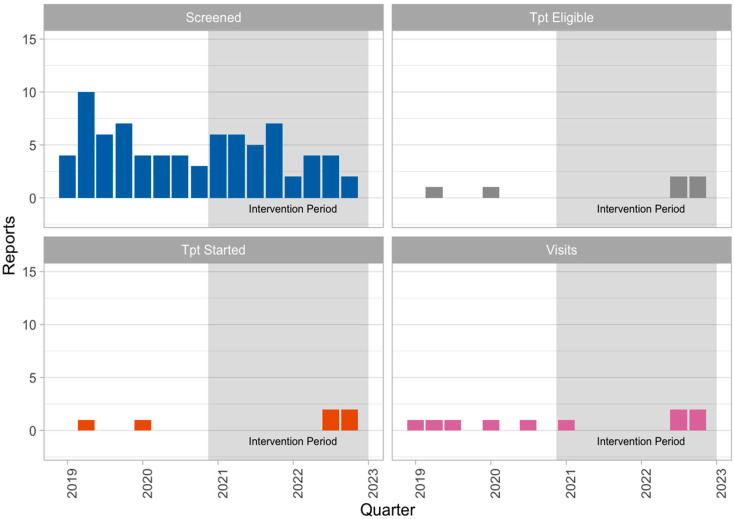
Number of reports where data were missing for each indicator.

The expected number of reports each quarter was 24 (eight facilities reporting monthly). Figure 1 shows the number of reports in each quarter where indicator data were unavailable for the following indicators: number of visits where a person was screened for TB; number of visits where a person was assessed as TPT eligible; number of persons started on TPT; and number of ART visits.

### 3.2. TB Screening Amongst PLHIV

**Figure 2:**
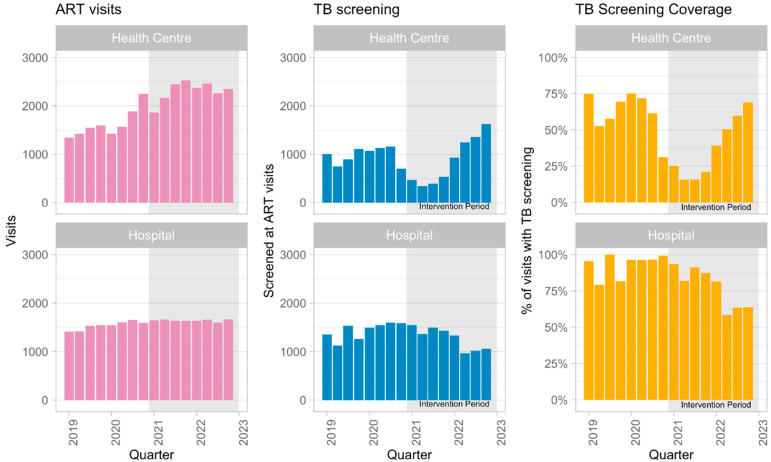
Total Number of TB screened and visits by health facilities type from SIHA by quarter.

A total of 31,586 visits by PLHIV to ART clinics in Yogyakarta City were recorded during the study period. Figure 2 shows the reported number of ART visits, the number of PLHIV visits with TB screening, and the screening coverage in each quarter by health facility type. The TB screening coverage was 78% (IQR 73–85%) in the baseline period and 55% (IQR 47–58%) in the intervention period. Despite the decrease, the screening coverage in health centers increased (Figure 1).

### 3.3. TB Preventive Therapy

**Figure 3:**
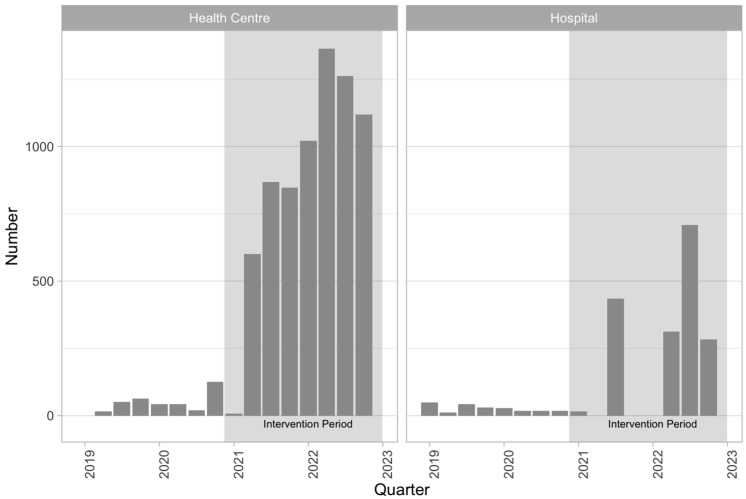
The number of PLHIV assessed and eligible for TPT each quarter.

In the pre-intervention period, there were 575 ART clinic visits at which a person living with HIV was assessed as eligible for TPT, compared to 8845 such visits in the intervention period. Figure 3 shows the number of patient visits at which the patient was assessed as eligible for TPT by quarter and health facility type. 

**Figure 4:**
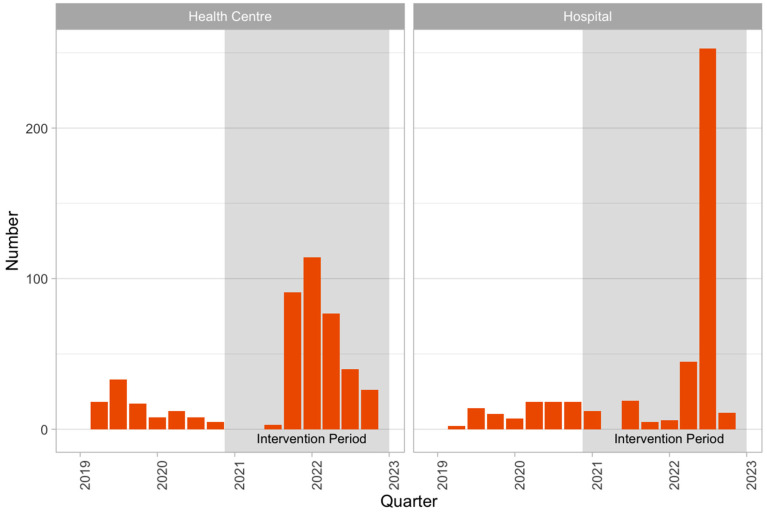
The number of PLHIV started on TPT each quarter by facility type.

In the pre-intervention period, 188 TPT initiations were reported compared to 702 in the intervention period. Figure 4 shows the number of patients started on TPT in each quarter by health facility type.

## 4. Discussion

The most notable change observed in this study was in the outcomes from TB screening among PLHIV: during the intervention period, a drastic increase in the number of visits at which patients were assessed as TPT-eligible was observed: 8845 in the intervention period compared to 575 in the baseline period—an increase of over 1500%. The number of PLHIV started on TPT also increased during the intervention period but at a lesser rate than the increase in eligibility: 188 PLHIV were started on ART during baseline, compared to 702 during the intervention. 

These increases relating to TPT among PLHIV were observed both in primary health centers and hospitals. TPT increase was very high in hospital services in Q3 2022. The increases likely reflect a change in practice where ART clinics more routinely assessed eligibility for TPT in the intervention period. Prior to the intervention, they focused only on screening for active TB. The observed increases are consistent with the results of other studies on operational scale-up of TB screening and TPT coverage among PLHIV, in which large increases in coverage of TPT have been achieved by interventions that address health service barriers to the provision of these services [27,28,29].

Due to the nature of reporting, the TPT eligibility indicator likely includes PLHIV who were repeatedly screened for TPT and repeatedly assessed as eligible before starting ART. However, the numbers may also reflect that there was a backlog of PLHIV who were not assessed as being TPT eligible during TB screening until ART services were more regularly assessing this at ART visits.

This study also confirms that ART facilities in Yogyakarta have routinely screened for tuberculosis (TB) in the PLHIV population, which is evident from the median quarterly screening coverage of 78% in the baseline period. At health centers, screening coverage was lower in the intervention period than in the baseline period. However, decreased coverage of TB screening among PLHIV was observed at the end of the baseline period, and coverage recovered during the intervention period.

Changes in screening coverage differed by health facility type: the drop and recovery in TB screening coverage were driven by screening coverage at primary health centers, whereas screening coverage at hospitals decreased slightly and gradually over the study period. In primary health centers during the final quarter of the pre-intervention period (Q4 2020), screening coverage fell to 31.2% compared to 52.6–75.1% in earlier quarters of the pre-intervention period and did not recover to above 50% until Q2 2022. The decrease in TB screening from late 2020 is likely to reflect the impact of the COVID-19 pandemic in Indonesia, which severely impacted healthcare services and care-seeking [16,25]. Other studies have reported disruptions in healthcare services, including HIV services, decreased visits to healthcare facilities, HIV testing, and drug distribution during the COVID-19 pandemic [16,23]. It is possible that during the COVID-19 pandemic, the interaction during visits was limited resulting in TB screening not being performed. This would be expected to result in a decreased proportion of ART visits where TB screening was conducted. It is likely that the impact of COVID-19 negated potential gains from the increase in TB screening.

The use of routinely reported surveillance data offers convenience in exploring whether any changes may have occurred during the intervention period. Specifically, the data does provide insight into drastic changes in patterns of TB screening and TPT among PLHIV by the eight health facilities included in the study. These eight facilities are the main providers of HIV services in Yogyakarta City. Given the need for improved coverage of TB screening and TPT among PLHIV in Indonesia, the results from this study indicate the possibility of achieving changes for improved coverage of TB services among PLHIV at ART clinics. Moreover, we have documented the model that the ZTB YY project used to strengthen TB services among PLHIV in Yogyakarta, which could provide insight into specific approaches that remain relevant for similar TB screening and TPT scale-up activities in other parts of Indonesia and other countries.

However, the data have some limitations. The quality and completeness of recording and reporting from health facilities in the data used for this study varied. Some reports were not submitted by health facilities and missing data were observed among the available reports. During data validation activities that were part of the support to the ART clinics, a ZTB nurse checked that TB screening and TPT eligibility from PLHIV participating in ACF were recorded in SIHA. Despite this, there was still some non-reporting of specific indicators. While the proportion of “missingness” was similar between the pre-intervention and the intervention periods, it is possible that the available data did not accurately reflect actual activity in TB screening and TPT initiation among PLHIV. The data from routine activities of HIV clinics outside of ZTB activity was not verified in detail. Strengthening of reporting will likely require support from health facilities. In addition, health facilities may wish to consider the periodic assessment of coverage of TB screening and TPT within their ART cohorts based on how many patients have been covered by these services rather than coverage among visits. In particular, the possibility of counting the same patient as TPT eligible multiple times limits the utility of routine report data in assessing TPT uptake and coverage.

## 5. Conclusions

During the intervention period, notable changes were observed in the assessment of TPT eligibility and increased numbers of PLHIV started on TPT. These data suggest that during the intervention period, a marked improvement occurred in whether and how health facilities assess PLHIV for TPT eligibility. Our study design, which relied on routine surveillance data, does not allow us to discern the extent to which Zero TB contributed to this shift. Nonetheless, the observed increases in TPT assessment and initiation represent important progress in integrating TB/HIV services and improving care for PLHIV. Other studies have highlighted the importance of addressing provider barriers, which is consistent with the model that the ZTB project employed [29,30,31]. As such, aspects of our model might have relevance for strategies to scale up TB screening and TPT coverage.

## Figures and Tables

**Table 1:** Routine TB–HIV Program and ZTB-supported models of TB–HIV services.

Model of TB–HIV Service	Routine TB–HIV Program Approach (Before 2021)	ZTB-Supported Model (2021–2022)
TB Screening Methods	TB symptom-based screening (W4SS) only Passive case finding.	TB symptom-based screening combined with referral to ZTB mobile CXR active case finding, e.g., inviting PLHIV to community-based mobile CXR.
Clinical decisions	Healthcare/hospital doctors.	Healthcare/hospital doctors supported by doctors from ZTB.
TB Prevention Therapy	6 months of Isoniazid (6H) for PLHIV without active TB.	6H for PLHIV without active TB for PLHIV on ART regimens that include nevirapine and protease inhibitors. Introduction of a short-term regimen of 3 months of isoniazid and rifapentine (3HP) for PLHIV on ART regimens without nevirapine and protease inhibitors.
Support and training for HIV Officer (Doctor, HIV Program Staff, RR officer)	Quarterly data validation meetings conducted by the health office and HIV program staff to ensure data on findings, ART initiation, and follow-up are reported in the HIV program recording system.	A dedicated ZTB nurse provided routine support to HIV program staff, and recording and reporting (RR) officers on TB–HIV collaboration, TPT, and recording and reporting. Technical assistance is needed to initiate and follow up on TPT and weekly records of all health facilities by the ZTB Nurse. Drug side effects, adherence, and TB symptoms were monitored during the follow-up visits.
Community engagement	Community involvement (Victory Plus, Vesta) in outreach to PLHIV to carry out routine ARV visits which included TB screening activities and initiation of TPT for those who meet the requirements.	Capacity building to the HIV community on basic TB–HIV and TPT information. Involvement of ZTB nurse in outreach to PLHIV for referral to screening.

**Table 2:** Availability of screening and TPT indicators from SIHA reports by study period.

SIHA Indicator	Pre-Intervention N = 192 ^1^	Intervention N = 192 ^1^	*p*-Value ^2^	Test Name
Visits			>0.9	Pearson’s Chi-squared test
Available	187 (97%)	187 (97%)		
Unavailable	5 (2.6%)	5 (2.6%)		
Screened			0.4	Pearson’s Chi-squared test
Available	150 (78%)	156 (81%)		
Unavailable	42 (22%)	36 (19%)		
TPT Eligible			0.7	Fisher’s exact test
Available	190 (99%)	188 (98%)		
Unavailable	2 (1.0%)	4 (2.1%)		
TPT Started			0.7	Fisher’s exact test
Available	190 (99%)	188 (98%)		
Unavailable	2 (1.0%)	4 (2.1%)		

^1^ n (%); ^2^ Pearson’s Chi-squared test; Fisher’s exact test.

## Data Availability

Due to data privacy concerns, data are not made publicly available. However, reasonable data requests may be granted through contacting the corresponding author.
